# Analysis of cardiac monitoring and safety data in patients initiating fingolimod treatment in the home or in clinic

**DOI:** 10.1186/s12883-019-1506-0

**Published:** 2019-11-15

**Authors:** Brandon Brown, Jamie L. Weiss, Scott Kolodny, Xiangyi Meng, Ian M. Williams, John A. Osborne

**Affiliations:** 1 Novartis Pharmaceuticals Corporation, One Health Plaza, East Hanover, NJ 07936 USA; 2 Oxford PharmaGenesis, Tubney Warren Barn, Tubney, Oxford, OX13 5QJ UK; 3 State of the Heart Cardiology, Plaza 1, Medical Pkwy. N Suite 103, Dallas, TX 75234 USA

**Keywords:** First-dose observation, Gilenya@home, Relapsing–remitting multiple sclerosis, Sphingosine 1-phosphate receptor, Bradycardia, Fingolimod, Safety, FDO

## Abstract

**Background:**

Fingolimod (Gilenya®) is approved for relapsing forms of multiple sclerosis in the USA. Owing to transient heart-rate effects when initiating fingolimod, eligible patients undergo precautionary baseline assessment and first-dose observation (FDO) for ≥6 h. Prior to 2014, FDO was undertaken only in clinics. As the FDO period is short, and fingolimod has accumulated evidence of a positive benefit:risk ratio, an in-home treatment-initiation program, Gilenya@Home, was developed to offer a convenient alternative.

**Methods:**

Cardiac parameters and adverse events (AEs) were recorded by healthcare professionals performing fingolimod FDOs in the US Gilenya@Home program or in US Gilenya Assessment Network clinics. Anonymized data were collated retrospectively from the first 34 months in the home setting and from 78 months in clinics; data are reported descriptively. Satisfaction with Gilenya@Home was rated by patients using a 7-item questionnaire that considered aspects such as ease of scheduling, courtesy, and competency.

**Results:**

Data were captured as part of standard care from 5573 patients initiating fingolimod in-home (October 2014 to July 2017) and from 15,025 patients initiating in-clinic (July 2010 to December 2016). In the Gilenya@Home questionnaire, 91.7% of 1848 respondents rated their overall satisfaction as “very good,” and 7.6% rated their satisfaction as “good.” AEs were reported for 30.7 and 32.6% of in-home and in-clinic patients, respectively. In total, 557 in-home (10.0%) and 398 in-clinic (2.6%) patients were monitored for > 6 h; 15 (0.3%) in-home and 129 (0.9%) in-clinic patients were transferred to an emergency room for overnight monitoring. The mean (standard deviation) heart rate (HR; bpm) pre-FDO was 74.8 (12.2) in-home and 74.2 (11.3) in-clinic; reduction in HR at 6 h postdose was 10.6 (12.0) and 6.3 (9.6), respectively. New-onset first-degree atrioventricular block was experienced by 132 (2.4%) in-home and 74 (0.5%) in-clinic patients, and Wenckebach (Mobitz type I) second-degree atrioventricular block by four (0.07%) and nine (0.1%) patients, with no cases of third-degree atrioventricular block.

**Conclusions:**

A substantial number of patients have initiated fingolimod at home, reporting very high levels of satisfaction. Gilenya@Home was as rigorous as the clinic setting in detecting cardiovascular events. Overall, FDO safety outcomes were similar with Gilenya@Home and in-clinic.

## Background

Fingolimod, a sphingosine 1-phosphate receptor (S1PR) agonist, is approved in the USA for the treatment of relapsing forms of multiple sclerosis (MS) [1]. As of 31 August 2018, it is estimated that more than 293,400 patients have been treated with fingolimod, corresponding to approximately 714,600 patient-years of exposure (data on file, Novartis Pharmaceuticals Corporation). Patients may experience transient effects on heart rate, arising from the actions of fingolimod at S1PRs found on atrial myocytes [2–6]; real-world evidence suggests that the first dose initiation is uneventful in most (> 90%) patients [7, 8]. Fingolimod is rapidly phosphorylated following absorption, and interactions between phosphorylated fingolimod and S1PRs lead to activation of the G-protein-coupled inwardly rectifying potassium channels causing a reduction in heart rate [6, 9, 10]. However, rapid cellular internalization and degradation of the fingolimod–S1PR complex mean that this effect is short-lived [1, 6, 11]. Downregulation and long-term suppression of these receptors are maintained by subsequent regular dosing with fingolimod, and so, when observed, the effects on heart rate are seen only at initiation rather than throughout the duration of fingolimod treatment [6, 11].

Owing to the potential transient effects of fingolimod on heart rate, all patients are observed for a minimum of 6 h following their first dose [1, 12]. For several years the first-dose observation (FDO) procedure was conducted only in medical facilities. The combination of a standardized baseline assessment protocol and the FDO procedure when initiating fingolimod has become well established under the guidance of healthcare professionals (HCPs) and at the many Gilenya Assessment Network sites in the USA. More recently, based on the history of use and the extensive safety data that have been gathered for fingolimod, as well as the relatively short duration of monitoring required following the first dose, the US Food and Drug Administration has allowed the introduction of an in-home FDO program for fingolimod initiation, called “Gilenya@Home.”

**Fig. 1:**
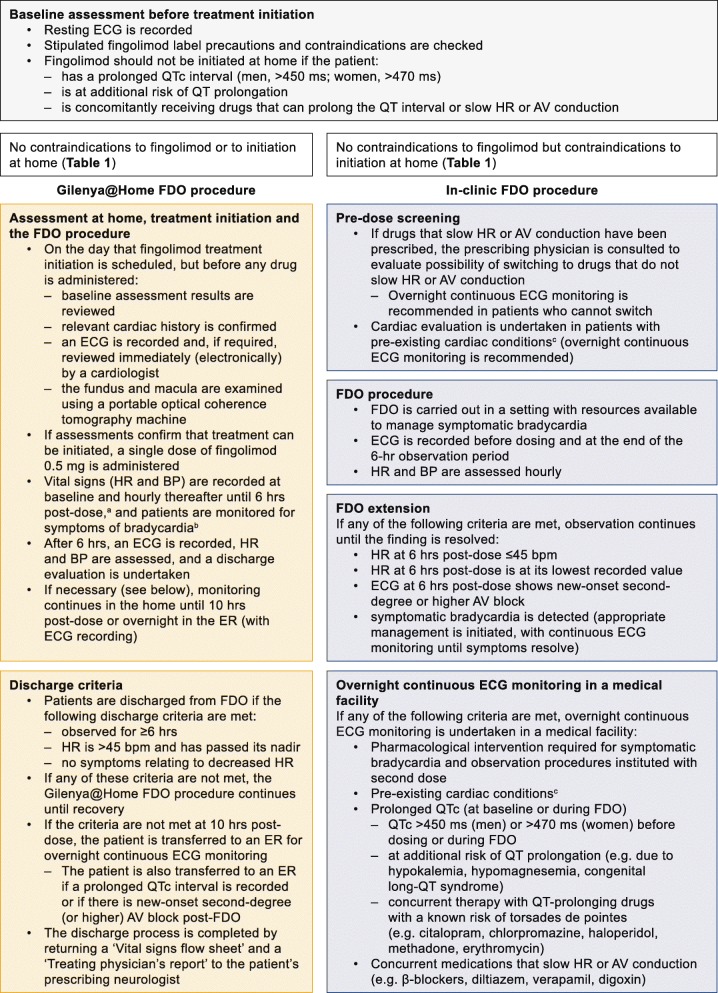
Overview of the Gilenya@Home FDO and in-clinic procedures. ^a^ Healthcare professionals were directed to measure vital signs (including blood pressure and heart rate) at baseline and then a minimum of once every hour throughout the rest of the procedure, although vital signs can be recorded more frequently, and for up to 10 h if needed. ^b^Syncope, near syncope, loss of consciousness, nausea, emesis, chest pain, or shortness of breath. ^c^Ischemic heart disease, history of myocardial infarction, congestive heart failure, history of cardiac arrest, cerebrovascular disease, uncontrolled hypertension, history of symptomatic bradycardia, history of recurrent syncope, severe untreated sleep apnoea, AV block, sinoatrial block. *AV* atrioventricular, *BP* blood pressure, *bpm* beats per minute, *ECG* electrocardiogram, *ER* emergency room, *FDO* first-dose observation, *HR* heart rate, *hr* hour, *ms* millisecond, *QTc* corrected QT interval

Fingolimod treatment initiation in the Gilenya@Home program is performed according to a defined protocol by an HCP and a medical assistant who attend the patient’s home, whereas fingolimod initiation in the clinic follows the standard protocol as per the prescribing information at a suitable medical facility [13]. The procedures for both programs are summarized in Fig. 1 (the full details of the Gilenya@Home program are provided in Appendix). Each patient undergoes a baseline assessment to evaluate their suitability for fingolimod treatment. This involves a resting electrocardiogram (ECG) recording and a review of medical history for any contraindications to fingolimod, or conditions or concomitant medications that preclude fingolimod initiation at home because of the need for overnight ECG monitoring (Tables 1 and 2). Based on their eligibility, patients may then choose to initiate fingolimod in clinic or at home (Fig. 1).

**Table 1:** Contraindications to in-home first-dose observation and to fingolimod in general

In-home first-dose observation contraindications	
Factors that contraindicate in-home fingolimod initiation, owing to the need for overnight electrocardiogram monitoring in a medical facility	Patients who poorly tolerate bradycardia or may experience serious heart rhythm disturbances, including those with:
	ischaemic heart disease
	history of myocardial infarction
	congestive heart failure
	history of cardiac arrest
	cerebrovascular disease
	uncontrolled hypertension
	history of symptomatic bradycardia
	history of recurrent syncope
	severe untreated sleep apnoea
	atrioventricular block
	sinoatrial heart block
General contraindications	
Factors that contraindicate treatment with fingolimod	Patients who in the preceding 6 months have experienced:
	myocardial infarction
	unstable angina
	stroke
	transient ischemic attack
	decompensated heart failure requiring hospitalization
	Class III/IV heart failure
	Patients with a history or presence of:
	Mobitz type II second- or third-degree atrioventricular block
	sick sinus syndrome (unless the patient has a pacemaker)
	a baseline corrected QT interval ≥ 500 ms
	Patients with cardiac arrhythmias requiring anti-arrhythmic treatment with Class Ia or class III anti-arrhythmic drugs (see Table 2)
	Patients who have experienced a hypersensitivity reaction to fingolimod (or its excipients) including rash, urticaria, or angioedema

**Table 2:** Drugs that contraindicate initiation of fingolimod

Contraindicate initiation of fingolimod in all settings	Contraindicate initiation of fingolimod in patient’s home but not in a medical facility	
Class Ia or class III antiarrhythmic drugs	Drugs that lower heart rate	Drugs that prolong QT interval with risk of torsades de pointes
Amiodarone	Calcium-channel blockers:	Anagrelide
Dofetilide	Diltiazem	Arsenic trioxide
Disopyramide	Verapamil	Azithromycin
Dronedarone	Cardioselective β-blockers:	Chloroquine
Ibutilide	Acebutolol	Chlorpromazine
Procainamide	Atenolol	Ciprofloxacin
Quinidine	Betaxolol	Citalopram
Sotalol	Bisoprolol	Cilostazol
	Esmolol	Clarithromycin
	Metoprolol	Cocaine
	Nebivolol	Donepezil
	β-Blockers with intrinsic sympathomimetic activity:	Droperidol
	Acebutolol	Erythromycin
	Carteolol	Escitalopram
	Penbutolol	Flecainide
	Pindolol	Fluconazole
	α-/β-Blockers:	Halofantrine
	Carvedilol	Haloperidol
	Labetalol	Levofloxacin
	Nonselective β-blockers:	Methadone
	Levobunolol	Moxifloxacin
	Metipranolol	Ondansetron
	Nadolol	Oxaliplatin
	Propranolol	Papaverine hydrochloride
	Sotalol	Pentamidine
	Timolol	Pimozide
	Other:	Propofol
	Adenosine	Sevoflurane
	Clonidine	Thioridazine
	Digoxin	Vandetanib
	Donepezil	
	Ivabradine	

Here we report a retrospective evaluation of safety data collected as part of the standard of care for patients initiating fingolimod in the Gilenya@Home program and in US Gilenya Assessment Network clinics. We also report findings from a patient questionnaire that examined satisfaction with the Gilenya@Home procedure.

## Methods

Fingolimod FDO procedures are conducted by an HCP and a medical assistant, and supervising HCPs have advanced cardiovascular life support training and appropriate training in the pharmacology of fingolimod and in the FDO procedure. Cardiac safety and adverse event (AE) data were collated retrospectively from anonymized patient records completed by HCPs in the Gilenya@Home program between 1 October 2014 and 31 July 2017 and in Gilenya Assessment Network clinics between 1 July 2010 and 31 December 2016. It should be noted the data collated are not from randomized controlled clinical trials. There were no recruitment processes per se and no predefined outcomes, and no power calculations were performed. Any patients prescribed fingolimod and referred to these programs by their physician were included, providing there were no contraindications in accordance with the product label or corresponding program (Table 1). Neither program was designed to capture demographic or baseline characteristic data; only age and sex data were available from the anonymized records. At all baseline assessments, the attending physician confirmed that patients were not receiving concomitant medications that would contraindicate the initiation of fingolimod, according to the relevant setting (Fig. 1, Table 2). Records of non-contraindicated concomitant medication usage were not available for either program.

Owing to the different program initiation contraindications (Table 1), Gilenya@Home and the Gilenya Assessment Network represent two non-overlapping, parallel, population-based, real-world datasets. As such, statistical analyses were considered not applicable. The safety data available from the anonymized records included heart rate at baseline and at the completion of FDO, occurrence and degree of atrioventricular (AV) block, AEs, whether extended monitoring was required, and whether the patient was transferred to an emergency room for overnight monitoring (Fig. 1). Use of concomitant medication was available only for the Gilenya Assessment Network dataset. AEs were coded using the Medical Dictionary for Regulatory Activities, and reported once per patient, including for patients in the Gilenya@Home program who initiated fingolimod multiple times within the study time frame. All summary safety findings are reported descriptively.

**Fig. 2:**
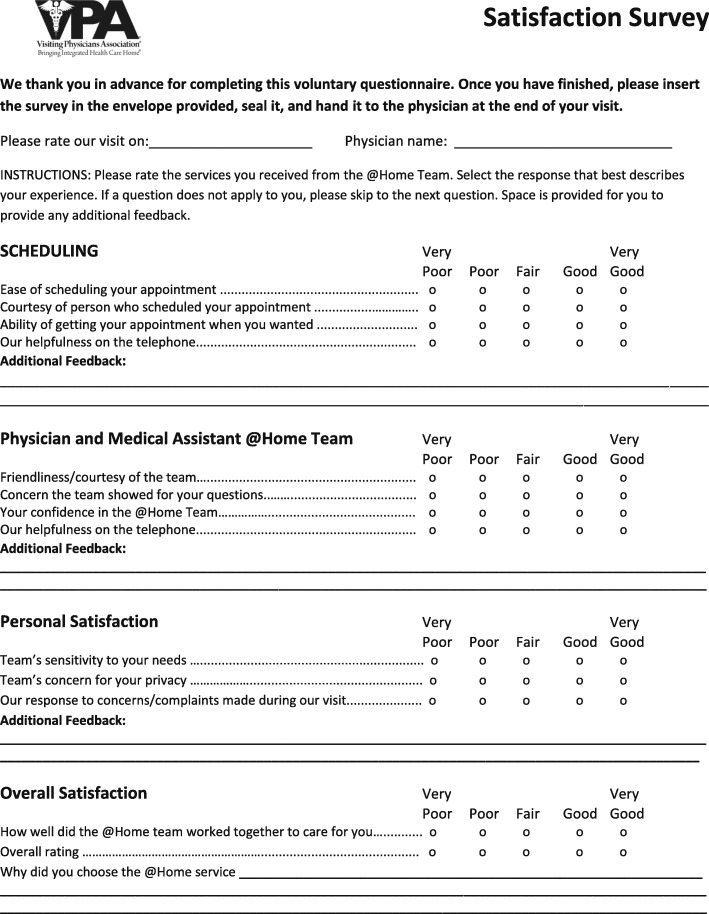
Patient satisfaction survey

Satisfaction with the Gilenya@Home process and with the attending medical teams was assessed using a survey (Fig. 2), which was completed by patients who had initiated fingolimod treatment under the Gilenya@Home program before February 2016. The survey form was sent by the Gilenya@Home administrators to the team providing the in-home procedure as part of their FDO kit. The attending HCP gave the survey to the patient at the end of the appointment. Patients could then complete the survey and return it directly to the Gilenya@Home administrators, independently of the on-site team. Once received, the administrators documented receipt of the survey form. The patient-satisfaction survey was designed to explore aspects such as the ease with which appointments could be scheduled and the helpfulness of those involved, the courtesy and perceived competency of the Gilenya@Home medical team in attendance, and the patient’s overall sense of satisfaction with the process. Patients provided a rating in answer to each question (“very good,” “good,” “fair,” “poor,” or “very poor”). Findings from the survey are reported descriptively.

Institutional review board (IRB) exemption was granted by Advarra IRB (Advarra, Columbia, MD) based on the study meeting the criteria: “Information, including information about the biospecimens, will be recorded in such a manner that the identity of the human subjects cannot readily be ascertained directly or through identifiers linked to the subjects, the investigator will not contact subjects, and the investigator will not reidentify subjects.”

## Results

### Patient recruitment

During the first 34 months of the Gilenya@Home program, a total of 5573 FDOs were performed (women, 4183 [75.1%]) for 5461 patients who initiated fingolimod at home; 112 FDOs were carried out for patients reinitiating fingolimod. Age data were available for 5060 visits. The mean (standard deviation [SD]) age of Gilenya@Home patients was 41.4 (10.5) years. Over a period of 78 months, 15,025 patients (women, 11,848 [78.9%]) initiated fingolimod in one of the Gilenya Assessment Network clinics in the USA. The mean (SD) age of in-clinic patients was 43.1 (11.1) years (data available for 14,873 visits).

Within the first 6 months of Gilenya@Home, the rate of fingolimod initiations in the program rose from 24 to 160 per month. Following this, the mean number of FDO observations performed within Gilenya@Home was 573 per quarter (data up to September 2017).

### Patient satisfaction

**Fig. 3:**
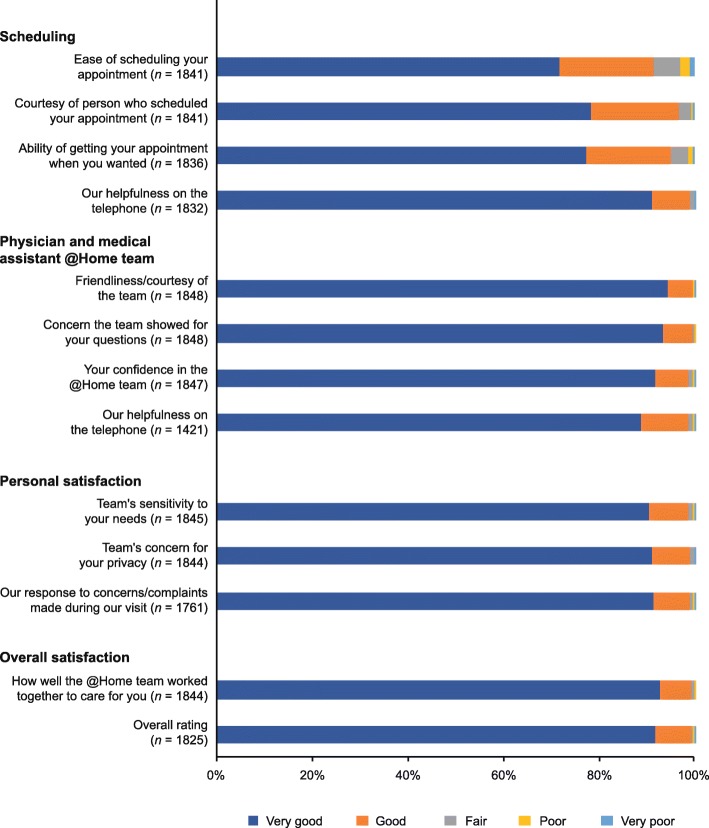
Patient satisfaction survey findings (*N* = 1848)

Of the 5573 FDO procedures performed between 1 October 2014 and 31 July 2017, 1848 patient surveys were returned and analysed (response rate, 33.2%). The survey results show that, for those aspects of the Gilenya@Home program that were evaluated, at least 90.0% of patients gave a satisfaction rating of “very good” or “good” for every category (Fig. 3). In terms of overall satisfaction, 99.3% of patients reported their satisfaction as “very good” (91.7%) or “good” (7.6%) (Fig. 3).

### Patient monitoring and safety

Of the 5573 visits that initiated fingolimod in home, 573 (10.3%) required extended monitoring beyond 6 h, and 15 (0.3%) were transferred to an emergency room to be monitored overnight. Among patients initiating fingolimod in clinic, 398 patients (2.6%) were monitored for more than 6 h, and 129 (0.9%) were monitored overnight in an emergency room.

AE data were available for 5460 of the patients initiating treatment at home; 1676 patients (30.7%) experienced an AE. The most common AEs were fatigue (*n* = 606; 11.1%), dizziness (*n* = 422; 7.73%), headache (*n* = 327; 5.99%), and somnolence (*n* = 140; 2.56%). No cardiac or vascular AEs of concern, including palpitations, bradycardia, cardiac flutter, or blood-pressure-related AEs, were experienced by > 2% of patients (Table 3). Among patients initiating fingolimod in clinic, 4899 (32.63%) experienced at least one AE. The most common AEs were fatigue (*n* = 760; 5.06%), dizziness (*n* = 684; 4.56%), headache (*n* = 616; 4.10%), decreased heart rate (*n* = 435; 2.90%), and somnolence (*n* = 346; 2.30%). Decreased heart rate was the only cardiac or vascular AE of interest experienced by > 2% of patients. All others, including palpitations, bradycardia, cardiac flutter, or blood-pressure-related AEs, affected < 1% of patients. The results from both programs followed a similar trend to the pooled findings from the phase 3 clinical trials of fingolimod [17], where the most common AEs also included fatigue, dizziness, headache, and somnolence, and all cardiac or vascular AEs of interest affected fewer than 1% of patients (Table 3). Full AE listings from the in-home and in-clinic datasets are presented in Additional file 1: Table S1 and Additional file 2: Table S2, respectively.

**Table 3:** First-dose observation AEs experienced by > 2% of patients in the Gilenya@Home, Gilenya Assessment Network, or pooled phase 3 trial populations, and cardiac- or vascular-related AEs of specific interest

	Gilenya@ Home (*n* = 5460)	Gilenya Assessment Network (*n* = 15,015)	Pooled phase 3 trials (*n* = 1212)
Preferred term	Frequency, *n* (%)	Frequency, *n* (%)	Frequency, *n* (%)
Total (any AE)	1676 (30.70)	4899 (32.63)	
Fatigue	606 (11.10)	760 (5.06)	17 (1.40)
Dizziness	422 (7.73)	684 (4.56)	21 (1.73)
Headache	327 (5.99)	616 (4.10)	41 (3.38)
Somnolence	140 (2.56)	346 (2.30)	7 (0.58)
Heart rate decreased	7 (0.13)	435 (2.90)	2 (0.17)
Nausea	77 (1.41)	168 (1.12)	25 (2.06)
Cardiac or vascular AEs of interest			
Chest discomfort	59 (1.08)	105 (0.70)	1 (0.08)
Palpitations	39 (0.71)	62 (0.41)	4 (0.33)
Feeling cold	33 (0.60)	39 (0.26)	1 (0.08)
Flushing	31 (0.57)	51 (0.34)	3 (0.25)
Feeling hot	29 (0.53)	44 (0.29)	1 (0.08)
Cardiac flutter	18 (0.33)	28 (0.19)	1 (0.08)
Chest pain	17 (0.31)	42 (0.28)	0
Sweating	11 (0.20)	4 (0.03)	0
AV block	9 (0.16)	41 (0.27)	3 (0.25)
Bradycardia	9 (0.16)	7 (0.05)	10 (0.83)^a^
Blood-pressure fluctuation	6 (0.11)	5 (0.03)	0
Peripheral coldness	6 (0.11)	4 (0.03)	0
Blood pressure increased	5 (0.09)	29 (0.19)	0
Sinus bradycardia	5 (0.09)	11 (0.07)	3 (0.25)
Hot flush	4 (0.07)	7 (0.05)	0
Hypertension	4 (0.07)	3 (0.02)	2 (0.17)
Heart rate increased	3 (0.05)	23 (0.15)	0
Heart rate irregular	2 (0.04)	14 (0.09)	0
Hypotension	2 (0.04)	7 (0.05)	1 (0.08)
Atrial fibrillation	1 (0.02)	0	0
Blood pressure decreased	1 (0.02)	48 (0.32)	0
Bundle branch block right	1 (0.02)	2 (0.01)	0
Raynaud’s phenomenon	1 (0.02)	0	0
Sinus tachycardia	1 (0.02)	0	0
Supraventricular extrasystoles	1 (0.02)	6 (0.04)	0
Syncope	1 (0.02)	0	0
ECG QT prolonged	0	56 (0.37)	1 (0.08)
ECG abnormal	0	52 (0.35)	0
ECG change	0	15 (0.10)	1 (0.08)
AV block second degree	0	9 (0.06)	1 (0.08)
ECG PR prolongation	0	4 (0.03)	0
Tachycardia	0	4 (0.03)	3 (0.25)
ECG ST segment elevation	0	2 (0.01)	1 (0.08)
Body temperature increased	0	1 (0.01)	0
Precordial exam finding	0	1 (0.01)	0
Presyncope	0	1 (0.01)	0

^a^Of these patients, 7 (0.6%) reported an associated symptom, 6 reported dizziness (5 mild, 1 moderate), and 1 reported moderate somnolence. Of the 5460 Gilenya@Home patients with AE data available, individual AEs were counted once per patient; for patients attending multiple first-dose observations, AEs were pooled from all visits. Of the 15,025 Gilenya Assessment Network clinic FDO procedures, AE data were available for 10,015 patients; 10 patients attended the clinic and began the FDO procedure but did not receive a dose of fingolimod. AEs reported for the pooled phase 3 trials include AEs in the first day following the first dose of fingolimod 0.5 mg in FREEDOMS [14], FREEDOMS II [15], and TRANSFORMS [16]. Please note, AEs of chest discomfort, chest pain, and temperature-related AEs were not confirmed as strictly cardiac- or vascular-related events but are included here for completeness. *AE* adverse event, *AV* atrioventricular, *ECG* electrocardiogram, *FDO* first-dose observation

The mean (SD) sitting heart rate before the first dose among patients initiating fingolimod in home was 74.8 (12.2) bpm, and 64.2 (12.4) bpm at 6 h after first dose (*n* = 5570), a reduction from baseline of 10.6 (12.0) bpm (Table 4; Gilenya@Home). Heart-rate data were available for 480 patients who were monitored beyond 6 h. In this subgroup, the mean (SD) heart rate at baseline was 72.4 (11.9) bpm, at 6 h 58.0 (9.0) bpm (a mean reduction from baseline of 14.4 [8.9] bpm), and after extended monitoring 63.9 (10.1) bpm (a mean reduction in heart rate from baseline of 8.5 [9.4] bpm). At 6 h, 25 patients (5.2%) in this subgroup had a heart rate of less than 45 bpm, and 465 patients (96.9%) had a heart rate below their baseline heart rate. By the end of extended monitoring, heart rate had increased in 427 of these 465 patients (91.8%). Among patients initiating fingolimod in clinic, the sitting heart rate before first dose was 74.2 (11.3) bpm, and the reduction from baseline at 6 h postdose was 6.3 (9.6) bpm (Table 4; Gilenya assessment network).

**Table 4:** Cardiovascular effects experienced by patients following first dose of fingolimod 0.5 mg

Event	Gilenya@ Home (*N* = 5573)	Gilenya Assessment Network (*N* = 15,025)	Pooled RCTs [17] (*n* = 1212)	FIRST [18] (*N* = 2415)		EPOC [19] (*N* = 976)	START [20] (*N* = 3951)^a^	Italian open-label trial [21] (*N* = 906)
				No PCCs (*n* = 2120)	PCCs (*n* = 295)			
Heart rate								
Maximum reduction, mean (SD), bpm	10.6 (12.0)	6.3 (9.6)	8.1 (8.1)	6.5 (NR)	7.4 (NR)	8.1 (8.3)	11.8 (8.5)	NR
Monitoring for more than 6 h	557 (10.0)	398 (2.6)	157 (13.0)	25 (2.6)^b^	15 (5.5)^b^	13 (1.3)	333 (8.4)	34 (3.8)
New-onset conduction abnormalities								
First-degree AV block	132 (2.4)	74 (0.5)	56 (4.7)^c^	0	0	16 (8.8)^d^	206 (5.8)	1 (0.1)
Second-degree AV block	4 (0.07)	9 (0.1)	2 (0.2)^c^	25 (1.2)	18 (6.1)	2 (0.2)^d^	78 (2.0)	2 (0.2)
Wenckebach (Mobitz type I) second-degree AV block	3 (0.05)	NR	2 (0.2)^c^	18 (0.9)	12 (4.1)	2 (0.2)^d^	60 (1.5)	2 (0.2)
2:1 s-degree AV block	0	NR	0^c^	7 (0.3)	6 (2.0)	NR	18 (0.5)	NR
Mobitz type II second-degree AV block	1 (0.02)	NR	0^c^	0	0	0^d^	0	NR

Data are *n* (%) unless stated otherwise. ^a^Interim data [20]. ^b^Events in the subgroup of patients monitored on site (*N* = 1219): no PCC, *n* = 948; PCC, *n* = 271. In total, 40 patients on site underwent extended monitoring, of whom 15 had PCCs [18]. ^c^Patients with electrocardiogram recordings, *n* = 1182; includes patients from the phase 3 RCTs of fingolimod [14–17]. ^d^New abnormalities among individuals receiving an electrocardiogram recording after more than 6 h of first-dose observation, *n* = 181 [19]. *AV* atrioventricular, *NR* not reported, *PCC* pre-existing cardiac condition, *RCT* randomized controlled trial, *SD* standard deviation

Onset of first-degree AV block during the 6-h monitoring period was recorded in 132 patients (2.4%) initiating fingolimod in home; none were transferred to an emergency room, but 18 received extended monitoring. Onset of second-degree AV block was observed in four patients (0.07%), one of whom had first-degree AV block before FDO and required extended monitoring in home but was not transferred to an emergency room. Three of these four patients required extended monitoring, and two were transferred to an emergency room to be monitored overnight. There were 74 cases (0.5%) of first-degree AV block and nine cases (0.1%) of second-degree AV block among patients initiating fingolimod in clinic. Of these 83 patients, most were discharged following FDO, four were transferred to an emergency room for overnight observation, and 16 were lost to follow-up. Third-degree AV block and torsade de pointes were not observed in either population (Table 4).

## Discussion

These cardiac safety data derived from two very large patient populations show that fingolimod initiation either in clinic or in the home under the Gilenya@Home program is associated with a good safety profile and appropriate surveillance. AEs reported for > 5% of patients in either setting were limited to fatigue, dizziness, and headache; cases of second-degree AV block were comparable to or lower than those reported in clinical trials; rates of extended monitoring were within the range of those reported in randomized controlled and postmarketing studies [17–21]; and no cases of third-degree block or torsade de pointes occurred. Overall, our survey found that respondents were very satisfied with Gilenya@Home.

The majority of patients starting fingolimod required monitoring only for the standard 6-h period following first dose. More patients received extended monitoring under the in-home program than did when initiating treatment in clinic (10.3% versus 2.6%, respectively). However, fewer patients observed at home were transferred to an emergency room for extended monitoring than when observation was conducted in clinic (0.3% versus 0.9%, respectively). When supervising the Gilenya@Home program, HCPs emphasize to patients that they must alert them to any side effects they experience. This measure is precautionary but necessary, given that the procedure is being conducted in a nonclinical environment. While the overall rates of AEs associated with the in-home program are broadly similar to those associated with fingolimod initiation in clinic, the increased vigilance probably leads to proportionally more patients receiving precautionary extended monitoring in home than in clinic. In contrast, proportionally fewer patients initiating treatment in home than in clinic were transferred to an emergency room for overnight monitoring after initiating fingolimod, although the difference between the groups was modest and, overall, the numbers transferred were small. It is unlikely that this difference is clinically significant. It is possible that by encouraging patients to report even mild side effects during the 6-h observation period, certain events were managed more promptly in the home setting than in clinic, perhaps mitigating issues that may lead to a requirement for overnight monitoring.

If the decision is made at 6 h that extended monitoring is needed, then patients are monitored in home up to a maximum of 10 h postdose before a decision is made whether to transfer patients to an emergency room for monitoring overnight. Procedural rather than symptomatic reasons to extend monitoring beyond 6 h are because heart rate is less than 45 bpm or because it has not passed its nadir. The reasons in each case for extending monitoring in about 10% of the in-home population and the timings of heart-rate nadir in these individuals are not known, but within this subgroup, only 5% had a heart rate less than 45 bpm at 6 h, and more than 95% had a heart rate at 6 h that was less than that at baseline, suggesting that heart-rate recovery may have been a very common reason to extend monitoring. The fact that about 95% of these patients with a reduced heart rate at 6 h had shown an increase in heart rate at the end of extended monitoring (and had therefore passed their heart-rate nadir) tends to corroborate the strategy that 6 h of FDO is sufficient for most patients and that a further 4 h provides sufficient additional time for recovery among nearly all patients. The mean heart rate at baseline was essentially the same in the in-home and in-clinic populations, but at 6 h the mean (±SD) heart rate dropped slightly further from baseline among those receiving fingolimod in home (10.6 ± 12.0 bpm) than in clinic (6.3 ± 9.6 bpm). However, considering the overlap in deviation of the two populations, it is unlikely to reflect a meaningful difference between the settings and, as there was no apparent effect on the rate of heart rate recovery, is probably not clinically significant.

Information regarding the incidence of cardiovascular side effects associated with initiation of fingolimod was in line with the data collected in the three pivotal phase 3 trials in relapsing–remitting MS (FREEDOMS [14] FREEDOMS II [15], and TRANSFORMS [16]) and in a pivotal phase 3 trial in primary progressive MS (INFORMS) [22]. Safety outcomes data from the three relapsing–remitting MS study populations have been published as a pooled analysis [17], and further data pertaining to fingolimod initiation have been published in several postmarketing studies, including FIRST [18], EPOC [19], and START [20], and from phase 4 trials conducted in Italy [21]; these findings are summarized in Table 4 alongside those from this retrospective analysis of fingolimod FDO in clinic and in the home. Published pooled data from 1212 patients in the FREEDOMS, FREEDOMS II, and TRANSFORMS trials who initiated fingolimod at the approved daily dose of 0.5 mg showed a maximum mean (SD) reduction in sitting heart rate of 8.1 (8.1) bpm [17]. In these and in other studies, the nadir in heart rate was generally reached 4–5 h after fingolimod was first ingested, and most patients experienced no symptoms associated with heart rate reduction. In the pooled phase 3 population, only 7 (0.6%) of the 1212 patients reported bradycardia with a symptom (dizziness, *n* = 6, 5 mild and 1 moderate; somnolence, *n* = 1, moderate) [17]. All of these events resolved without intervention. Rates of AEs, and also rates of extended monitoring, are generally lower than those seen in our analysis of the in-home population, but as was noted when comparing in-home and in-clinic findings, this may simply reflect the heightened precautionary measures associated with treatment initiation in a nonclinical setting. Overall, the AE profile at fingolimod initiation was consistent across clinical settings, and that the vast majority of cardiac or vascular AEs occurred in far fewer than 1% of patients.

Indeed, a small number of cases of second-degree AV block were recorded among patients initiating fingolimod in home or in clinic (13 in total among more than 20,000 patients). This rate is consistent with, but lower than, that seen in the pooled phase 3 trials, in which ECG recordings revealed two patients with Wenckebach (Mobitz type I) second-degree AV block [17]. Event rates observed in the postmarketing clinical studies were generally similar, although rates of conduction abnormalities were noticeably higher in patients with pre-existing cardiac conditions who participated in the FIRST trial. These postmarketing studies included patients receiving β-blockers or calcium-channel blockers, so their populations might be considered more representative of the real-world population than those in the controlled trials. The fact that rates of second-degree conduction abnormalities were lower than this in the in-home and in-clinic populations, both of which actually comprise the general MS population, should reassure clinicians that the procedures in place to initiate fingolimod in these settings are both sufficient and robust.

Provision of the FDO procedure at a patient’s home offers an alternative to initiating treatment at a medical facility, and most patients were very satisfied with the in-home program, all aspects evaluated being rated as “very good” or “good” by at least 89% of respondents. The ability to provide the FDO procedure at home offers a number of potential benefits over fingolimod initiation in a medical facility. With most forms of commercially available US health insurance, FDO is provided at no cost to patients in both the in-home and in-clinic settings [23]. It is estimated that performing FDO in clinics is less expensive for payers than in-home FDO, owing to the ease of administrating procedures on set clinic days with a dedicated clinical team, although this puts the burden on patients to fit into the clinic schedule. The in-home FDO procedure has made fingolimod treatment initiation more convenient for patients by comparison. Individuals who are employed would otherwise have to be absent from work to spend at least a day in a medical facility; furthermore, patients with limited mobility are helped by not having to travel to such a facility, which in some cases may be a considerable distance away. Indeed, the positive response from the satisfaction survey suggests that these benefits are considerable, and important to the wellbeing of patients. The in-home procedure may also help to relieve pressure in clinics, especially as weekend scheduling of appointments is not always offered by such medical facilities. The rigorous assessment process undertaken both before and during treatment initiation ensures that patient safety is not compromised when fingolimod treatment is initiated at home.

## Conclusions

The data presented provide evidence that the monitoring outcomes were similar overall between Gilenya@Home and in-clinic programs. Because Gilenya@Home and the Gilenya Assessment Network represent two parallel, non-overlapping, population-based datasets, there are a number of limitations to be considered. The data reported here are descriptive, and only information directly relevant to the final FDO outcome was captured. As such, a more detailed presentation of heart rate or blood pressure changes, including those at nadir, was not possible. No data were available in terms of disease history, comorbidities, and concomitant medication use or baseline clinical and demographic data. Therefore, definitive conclusions cannot be made on the relative composition of the two patient populations, or how they compare with those assessed in randomized controlled trials or postmarketing studies.

However, it should be noted that Gilenya@Home and the Gilenya Assessment Network represent real-world patient populations, with patients entering either program according to the actual prescribing decisions made by physicians. Owing to the large number of patients included, it is reasonable to assume that the patient populations reported here approximate the typical spectrum of patients in the USA with relapsing forms of MS who were prescribed fingolimod during the period assessed by this study.

Overall, the data presented suggest that for patients who fulfil the eligibility criteria at the baseline assessment and who have no contraindications to outpatient FDO, the safety profile of fingolimod treatment initiation at home is broadly similar to initiation in clinic. Since its commencement in October 2014, the data confirm that the in-home initiation of fingolimod has been widely adopted by HCPs and patients, with the majority of patients reporting a high level of patient satisfaction while potentially relieving pressure in clinics. In addition, the safety profile of Gilenya@Home suggests that the processes followed by supervising HCPs and medical assistants prior to and during initiation are appropriate to this clinical setting, and may provide assurance to prescribing physicians that their patients are subject to robust obligatory procedures, precautions, and safeguards during in-home fingolimod initiation.

### Supplementary information

**Additional file 1: Table S1.** Full listing of AEs from the Gilenya@Home dataset, in order of decreasing frequency.

**Additional file 2: Table S2.** Full listing of AEs from the Gilenya Assessment Network clinics dataset, in order of decreasing frequency.

## Data Availability

All available AE data are included in the additional tables. The datasets used and analysed during the current study are available from the corresponding author on reasonable request.

## Appendix

### Gilenya@Home procedures for fingolimod initiation

A healthcare professional and a medical assistant attend the patient’s home on the day of treatment initiation; supervising healthcare professionals have advanced cardiovascular life-support training and advanced training in the pharmacology of fingolimod and the first-dose observation procedure, and may be physicians, physician assistants, or nurse practitioners. Before initiating fingolimod, all patients must undergo baseline assessment including recordings and clinical laboratory measurements, which can also be undertaken as part of the Gilenya@Home program.

### Baseline assessment before treatment initiation

A resting electrocardiogram is recorded, and the precautions and contraindications stipulated in the fingolimod label are checked. Certain parameters require monitoring in a medical facility during fingolimod initiation and therefore preclude treatment initiation in a patient’s home (Table 2). Owing to a potential need for overnight electrocardiogram monitoring, fingolimod should not be initiated in the patient’s home if a patient has a prolonged corrected QT interval (men, > 450 ms; women, > 470 ms), is at additional risk for QT prolongation (e.g. because of hypokalemia, hypomagnesemia, or congenital long-QT syndrome), or is concomitantly receiving either drugs that can prolong the QT interval with a known risk of torsades de pointes or drugs that slow heart rate or atrioventricular conduction (Table 3).

### In-home assessment, treatment initiation, and the first-dose observation procedure

On the day scheduled for fingolimod initiation, and before any drug is administered, the results of the baseline assessment are reviewed, relevant cardiac history is confirmed, and an electrocardiogram recording is performed, irrespective of whether it formed part of the baseline assessment. If required, the electrocardiogram recording can be sent electronically to a cardiologist for immediate review before proceeding with treatment initiation. Another assessment that must take place before fingolimod treatment can be initiated is examination of the fundus and macula. To enable this assessment to take place in the setting of the patient’s home, the use of portable optical coherence tomography machines (OptiVue, Oregon, OH) has been piloted and recently expanded within the Gilenya@Home program.

If these assessments confirm that treatment can be initiated, a single 0.5 mg dose of fingolimod is administered, and the in-home first-dose observation procedure is initiated with recording of administration time and the patient’s baseline heart rate and blood pressure. Vital signs (heart rate and blood pressure) are recorded hourly (or more frequently if indicated) during the entire 6-h observation period and for up to 10 h if necessary. Patients are also monitored for symptoms of bradycardia (syncope, near syncope, loss of consciousness, nausea, emesis, chest pain, or shortness of breath). At the end of the observation period, an electrocardiogram is recorded, vital signs are measured, and a discharge evaluation is undertaken. If a patient experiences symptomatic bradycardia with a heart rate of less than 50 bpm, 12-lead electrocardiogram monitoring is commenced, and vital signs and oxygen saturation are recorded every 30 min. If the patient experiences bradycardia and signs of poor perfusion, and the supervising healthcare professional deems it necessary, a single intravenous dose of atropine 0.5 mg is administered, the prescribing healthcare professional is notified, and the patient is transferred immediately to an emergency room for continuous electrocardiogram monitoring over 24 h.

### Discharge criteria

Patients meet the discharge criteria (i.e. they are discharged from first-dose observation) if they have been observed for a minimum of 6 h, their heart rate is > 45 bpm and has passed its nadir, and they are asymptomatic for a decreased heart rate. If any of these criteria are unmet, the in-home first-dose observation procedure continues until recovery. If these criteria are not met at 10 h, a prolonged QTc interval is recorded, or if there is new-onset second-degree (or higher) AV block at electrocardiogram post-first-dose observation, patients are transferred to an emergency room for continuous electrocardiogram monitoring overnight. The discharge process is completed by returning a “Vital Signs Flow Sheet” and a “Treating Physician Report” to the patient’s prescribing neurologist; if the first-dose observation procedure is cancelled, these forms must also be completed, including the rationale for cancellation, and returned to the prescribing healthcare professional. Before discharge, patients and caregivers must be made aware that milder effects on heart rate than those observed during the first-dose observation procedure may persist during the first 4 weeks of treatment, and that they therefore must be alert to possible symptoms. Patients are also provided with counseling information and a fingolimod medication guide and are asked to complete and return a “Gilenya@Home First-Dose Survey.”

## Abbreviations

AE: Adverse event
AV: Atrioventricular
BP: Blood pressure
bpm: Beats per minute
ECG: Electrocardiogram
ER: Emergency room
FDO: First-dose observation
HCP: Healthcare professionals
HR: Heart rate
IRB: Institutional review board
MS: Multiple sclerosis
NR: Not reported
PCC: Pre-existing cardiac condition
QTc: Corrected QT interval
RCT: Randomized controlled trial
S1PR: Sphingosine 1-phosphate receptor
SD: Standard deviation
