# Correction to: Analysis of cardiac monitoring and safety data in patients initiating fingolimod treatment in the home or in clinic

**DOI:** 10.1186/s12883-019-1568-z

**Published:** 2019-12-21

**Authors:** Brandon Brown, Jamie L. Weiss, Scott Kolodny, Xiangyi Meng, Ian M. Williams, John A. Osborne

**Affiliations:** 1 Novartis Pharmaceuticals Corporation, One Health Plaza, East Hanover, NJ 07936 USA; 2 Oxford PharmaGenesis, Tubney Warren Barn, Tubney, Oxford, OX13 5QJ UK; 3 State of the Heart Cardiology, Plaza 1, Medical Pkwy. N Suite 103, Dallas, TX 75234 USA

**Correction to: BMC Neurol (2019) 19:287**

**https://doi.org/10.1186/s12883-019-1506-0**

Following publication of the original article [1], an error was noted regarding the year found in the paragraph of the Background section.

The sentence should read ‘As of 31 August **2019**, it is estimated that more than 293,400 patients have been treated with fingolimod, corresponding to approximately 714,600 patient-years of exposure (data on file, Novartis Pharmaceuticals Corporation)’.

## References

[1] Brown B, Weiss JL, Kolodny S, Meng X, Williams IM, Osborne JA (2019). Analysis of cardiac monitoring and safety data in patients initiating fingolimod treatment in the home or in clinic. BMC Neurol.

