# Supplementary material for: Analysis of cardiac monitoring and safety data in patients initiating fingolimod treatment in the home or in clinic
Source: BMC Neurol. 2019 Nov 15;19:287. doi: 10.1186/s12883-019-1506-0 (PMC6857316; doi:10.1186/s12883-019-1506-0)
Supplement: Supplementary file 1 — Additional file 1: Table S1. Full listing of AEs from the Gilenya@Home dataset, in order of decreasing frequency. [file 12883_2019_1506_MOESM1_ESM.docx]

Additional file 1: **Table S1.** Full listing of AEs from the Gilenya@Home dataset, in order of decreasing frequency

| Preferred term | Frequency (*n*) | Percentage of patients |
| --- | --- | --- |
| Total (any AE) | 1676 | 30.70 |
| Fatigue | 606 | 11.10 |
| Dizziness | 422 | 7.73 |
| Headache | 327 | 5.99 |
| Somnolence | 140 | 2.56 |
| Nausea | 77 | 1.41 |
| Chest discomfort | 59 | 1.08 |
| Disorientation | 53 | 0.97 |
| Nervousness | 53 | 0.97 |
| Paresthesia | 50 | 0.92 |
| Anxiety | 47 | 0.86 |
| Palpitations | 39 | 0.71 |
| Euphoric mood | 38 | 0.70 |
| Mood altered | 37 | 0.68 |
| Feeling cold | 33 | 0.60 |
| Flushing | 31 | 0.57 |
| Dysgeusia | 29 | 0.53 |
| Feeling hot | 29 | 0.53 |
| Feeling abnormal | 26 | 0.48 |
| Asthenia | 23 | 0.42 |
| Dyspnea | 23 | 0.42 |
| Hunger | 22 | 0.40 |
| Hypoesthesia | 20 | 0.37 |
| Dyspepsia | 19 | 0.35 |
| Cardiac flutter | 18 | 0.33 |
| Feeling jittery | 18 | 0.33 |
| Chest pain | 17 | 0.31 |
| Vision blurred | 14 | 0.26 |
| Dry mouth | 13 | 0.24 |
| Pain | 13 | 0.24 |
| Diarrhea | 12 | 0.22 |
| Lethargy | 12 | 0.22 |
| Pain in extremity | 12 | 0.22 |
| Balance disorder | 11 | 0.20 |
| Sweating | 11 | 0.20 |
| Pruritus | 10 | 0.18 |
| AV block | 9 | 0.16 |
| Back pain | 9 | 0.16 |
| Bradycardia | 9 | 0.16 |
| Cough | 8 | 0.15 |
| Energy increased | 8 | 0.15 |
| Malaise | 8 | 0.15 |
| Musculoskeletal pain | 8 | 0.15 |
| Sluggishness | 8 | 0.15 |
| Head discomfort | 7 | 0.13 |
| Heart rate decreased | 7 | 0.13 |
| Migraine | 7 | 0.13 |
| Muscular weakness | 7 | 0.13 |
| Tremor | 7 | 0.13 |
| Abdominal pain | 6 | 0.11 |
| Abdominal pain upper | 6 | 0.11 |
| Blood-pressure fluctuation | 6 | 0.11 |
| Muscle tightness | 6 | 0.11 |
| Peripheral coldness | 6 | 0.11 |
| Incomplete AE data/not recorded | 6 | 0.11 |
| Sensation of heaviness | 6 | 0.11 |
| Abdominal discomfort | 5 | 0.09 |
| Blood pressure increased | 5 | 0.09 |
| Burning sensation | 5 | 0.09 |
| Dehydration | 5 | 0.09 |
| Muscoskeletal stiffness | 5 | 0.09 |
| Sinus bradycardia | 5 | 0.09 |
| Stomach discomfort | 5 | 0.09 |
| Thirst | 5 | 0.09 |
| Vertigo | 5 | 0.09 |
| Cognitive disorder | 4 | 0.07 |
| Depressed mood | 4 | 0.07 |
| Hot flush | 4 | 0.07 |
| Hypertension | 4 | 0.07 |
| Muscle spasms | 4 | 0.07 |
| Arthralgia | 3 | 0.05 |
| Chills | 3 | 0.05 |
| Dizziness postural | 3 | 0.05 |
| Heart rate increased | 3 | 0.05 |
| Tension headache | 3 | 0.05 |
| Throat tightness | 3 | 0.05 |
| Tinnitus | 3 | 0.05 |
| Asthenopia | 2 | 0.04 |
| Confusional state | 2 | 0.04 |
| Discomfort | 2 | 0.04 |
| Disturbance in attention | 2 | 0.04 |
| Dysarthria | 2 | 0.04 |
| Ear congestion | 2 | 0.04 |
| Emotional disorder | 2 | 0.04 |
| Eye pain | 2 | 0.04 |
| Eye pruritus | 2 | 0.04 |
| Feeling of body temperature change | 2 | 0.04 |
| Gait disturbance | 2 | 0.04 |
| Heart rate irregular | 2 | 0.04 |
| Hypersensitivity | 2 | 0.04 |
| Hypoacusis | 2 | 0.04 |
| Hypoesthesia facial | 2 | 0.04 |
| Hypotension | 2 | 0.04 |
| Lacrimation increased | 2 | 0.04 |
| Myalgia | 2 | 0.04 |
| Nasal congestion | 2 | 0.04 |
| Nasopharyngitis | 2 | 0.04 |
| Neck pain | 2 | 0.04 |
| Nicotine dependence | 2 | 0.04 |
| Photophobia | 2 | 0.04 |
| Rash | 2 | 0.04 |
| Rhinorrhea | 2 | 0.04 |
| Sensation of pressure | 2 | 0.04 |
| Stress | 2 | 0.04 |
| Visual acuity increased | 2 | 0.04 |
| Vomiting | 2 | 0.04 |
| Abdominal pain lower | 1 | 0.02 |
| Abnormal sensation in eye | 1 | 0.02 |
| Altered state of consciousness | 1 | 0.02 |
| Aphonia | 1 | 0.02 |
| Atrial fibrillation | 1 | 0.02 |
| Blood pressure decreased | 1 | 0.02 |
| Bundle branch block right | 1 | 0.02 |
| Coordination abnormal | 1 | 0.02 |
| Depersonalization | 1 | 0.02 |
| Diplopia | 1 | 0.02 |
| Dysphagia | 1 | 0.02 |
| Ear discomfort | 1 | 0.02 |
| Erythema | 1 | 0.02 |
| Eye irritation | 1 | 0.02 |
| Eye-movement disorder | 1 | 0.02 |
| Facial pain | 1 | 0.02 |
| Fall | 1 | 0.02 |
| Flatulence | 1 | 0.02 |
| Frequent bowel movements | 1 | 0.02 |
| Hyperaesthesia | 1 | 0.02 |
| Hyperpnea | 1 | 0.02 |
| Hypoesthesia oral | 1 | 0.02 |
| Hypoglycemia | 1 | 0.02 |
| Hypopnea | 1 | 0.02 |
| Improved back pain | 1 | 0.02 |
| Influenza-like illness | 1 | 0.02 |
| Joint swelling | 1 | 0.02 |
| Laryngitis | 1 | 0.02 |
| Libido increased | 1 | 0.02 |
| Lip swelling | 1 | 0.02 |
| Mobility increased | 1 | 0.02 |
| Muscle twitching | 1 | 0.02 |
| Muscoskeletal discomfort | 1 | 0.02 |
| Oropharyngeal pain | 1 | 0.02 |
| Piloerection | 1 | 0.02 |
| Pregnancy | 1 | 0.02 |
| Pyrexia | 1 | 0.02 |
| Raynaud’s phenomenon | 1 | 0.02 |
| Restlessness | 1 | 0.02 |
| Salivary hypersecretion | 1 | 0.02 |
| Sensory loss | 1 | 0.02 |
| Sinus congestion | 1 | 0.02 |
| Sinus headache | 1 | 0.02 |
| Sinus tachycardia | 1 | 0.02 |
| Skin discoloration | 1 | 0.02 |
| Skin lesion | 1 | 0.02 |
| Skin tightness | 1 | 0.02 |
| Sleep disorder | 1 | 0.02 |
| Sneezing | 1 | 0.02 |
| Staphylococcal infection | 1 | 0.02 |
| Strabismus | 1 | 0.02 |
| Stress at work | 1 | 0.02 |
| Supraventricular extrasystoles | 1 | 0.02 |
| Syncope | 1 | 0.02 |
| Tension | 1 | 0.02 |
| Throat irritation | 1 | 0.02 |
| Urticaria | 1 | 0.02 |
| Visual acuity reduced | 1 | 0.02 |
| Wheezing | 1 | 0.02 |

**Total number of Gilenya@Home patients with available AE data; *n* = 5460. Individual AEs were counted once per patient. For patients attending multiple first-dose observations, AEs were pooled from all visits. *AE* adverse event; *AV* atrioventricular.**
