# Supplementary material for: Analysis of cardiac monitoring and safety data in patients initiating fingolimod treatment in the home or in clinic
Source: BMC Neurol. 2019 Nov 15;19:287. doi: 10.1186/s12883-019-1506-0 (PMC6857316; doi:10.1186/s12883-019-1506-0)
Supplement: Supplementary file 2 — Additional file 2: Table S2. Full listing of AEs from the Gilenya Assessment Network clinics dataset, in order of decreasing frequency. [file 12883_2019_1506_MOESM2_ESM.docx]

Additional file 2: **Table S2.** Full listing of AEs from the Gilenya Assessment Network clinics dataset, in order of decreasing frequency

| Preferred term | Frequency (*n*) | Percentage of patients |
| --- | --- | --- |
| Total (any AE) | 4899 | 32.627 |
| Fatigue | 760 | 5.062 |
| Dizziness | 684 | 4.555 |
| Headache | 616 | 4.103 |
| Heart rate decreased | 435 | 2.897 |
| Somnolence | 346 | 2.304 |
| Nausea | 168 | 1.119 |
| Incomplete AE data/not recorded | 125 | 0.833 |
| Chest discomfort | 105 | 0.699 |
| Paresthesia | 77 | 0.513 |
| Dysgeusia | 65 | 0.433 |
| Palpitations | 62 | 0.413 |
| ECG QT prolonged | 56 | 0.373 |
| ECG abnormal | 52 | 0.346 |
| Flushing | 51 | 0.340 |
| Blood pressure decreased | 48 | 0.320 |
| Dyspnea | 47 | 0.313 |
| Feeling hot | 44 | 0.293 |
| Vision blurred | 44 | 0.293 |
| Disorientation | 43 | 0.286 |
| Chest pain | 42 | 0.280 |
| AV block | 41 | 0.273 |
| Feeling cold | 39 | 0.260 |
| Pain in extremity | 36 | 0.240 |
| Asthenia | 33 | 0.220 |
| Anxiety | 31 | 0.206 |
| Blood pressure increased | 29 | 0.193 |
| Cardiac flutter | 28 | 0.186 |
| Dyspepsia | 28 | 0.186 |
| Back pain | 27 | 0.180 |
| Feeling abnormal | 27 | 0.180 |
| Hypoesthesia | 25 | 0.167 |
| Balance disorder | 24 | 0.160 |
| Heart rate increased | 23 | 0.153 |
| Feeling jittery | 22 | 0.147 |
| Tremor | 22 | 0.147 |
| Pruritus | 20 | 0.133 |
| Stomach discomfort | 20 | 0.133 |
| Nervousness | 19 | 0.127 |
| Diarrhea | 17 | 0.113 |
| ECG change | 15 | 0.100 |
| Heart rate irregular | 14 | 0.093 |
| Muscle spasms | 14 | 0.093 |
| Pain | 14 | 0.093 |
| Dry mouth | 13 | 0.087 |
| Visual impairment | 13 | 0.087 |
| Vomiting | 12 | 0.080 |
| Dizziness postural | 11 | 0.073 |
| Sinus bradycardia | 11 | 0.073 |
| Sensation of heaviness | 10 | 0.067 |
| AV block second degree | 9 | 0.060 |
| Head discomfort | 9 | 0.060 |
| Hypoesthesia facial | 9 | 0.060 |
| Musculoskeletal discomfort | 9 | 0.060 |
| Neck pain | 9 | 0.060 |
| Abdominal pain upper | 8 | 0.053 |
| Mood altered | 8 | 0.053 |
| Muscle tightness | 8 | 0.053 |
| Abdominal pain | 7 | 0.047 |
| Bradycardia | 7 | 0.047 |
| Chills | 7 | 0.047 |
| Hot flush | 7 | 0.047 |
| Hypotension | 7 | 0.047 |
| Edema peripheral | 7 | 0.047 |
| Sluggishness | 7 | 0.047 |
| Vertigo | 7 | 0.047 |
| Cough | 6 | 0.040 |
| Eye pain | 6 | 0.040 |
| Malaise | 6 | 0.040 |
| Musculoskeletal pain | 6 | 0.040 |
| Musculoskeletal stiffness | 6 | 0.040 |
| Myalgia | 6 | 0.040 |
| Pollakiuria | 6 | 0.040 |
| Tinnitus | 6 | 0.040 |
| Ventricular extrasystoles | 6 | 0.040 |
| Abdominal discomfort | 5 | 0.033 |
| Blood-pressure fluctuation | 5 | 0.033 |
| Burning sensation | 5 | 0.033 |
| Erythema | 5 | 0.033 |
| Hypoesthesia oral | 5 | 0.033 |
| Rash | 5 | 0.033 |
| Abdominal pain lower | 4 | 0.027 |
| Abnormal sensation in eye | 4 | 0.027 |
| Asthenopia | 4 | 0.027 |
| ECG PR prolongation | 4 | 0.027 |
| Euphoric mood | 4 | 0.027 |
| Peripheral coldness | 4 | 0.027 |
| Photophobia | 4 | 0.027 |
| Respiratory disorder | 4 | 0.027 |
| Restlessness | 4 | 0.027 |
| Sweating | 4 | 0.027 |
| Tachycardia | 4 | 0.027 |
| Throat irritation | 4 | 0.027 |
| Throat tightness | 4 | 0.027 |
| Diplopia | 3 | 0.020 |
| Emotional disorder | 3 | 0.020 |
| Feeling of body-temperature change | 3 | 0.020 |
| Flatulence | 3 | 0.020 |
| Hunger | 3 | 0.020 |
| Hypertension | 3 | 0.020 |
| Lethargy | 3 | 0.020 |
| Muscle twitching | 3 | 0.020 |
| Nasopharyngitis | 3 | 0.020 |
| Rhinorrhea | 3 | 0.020 |
| Sensation of foreign body | 3 | 0.020 |
| Sinus headache | 3 | 0.020 |
| Vitreous floaters | 3 | 0.020 |
| Abdominal distension | 2 | 0.013 |
| Arthralgia | 2 | 0.013 |
| Blepharospasm | 2 | 0.013 |
| Bundle branch block right | 2 | 0.013 |
| Depressed mood | 2 | 0.013 |
| Discomfort | 2 | 0.013 |
| Dry throat | 2 | 0.013 |
| Dysarthia | 2 | 0.013 |
| Ear discomfort | 2 | 0.013 |
| ECG ST segment elevation | 2 | 0.013 |
| Eye pruritus | 2 | 0.013 |
| Gait disturbance | 2 | 0.013 |
| Influenza-like illness | 2 | 0.013 |
| Joint pain | 2 | 0.013 |
| Migraine | 2 | 0.013 |
| Muscular weakness | 2 | 0.013 |
| Nasal congestion | 2 | 0.013 |
| Oropharyngeal pain | 2 | 0.013 |
| Pain in jaw | 2 | 0.013 |
| Swelling face | 2 | 0.013 |
| Vertigo positional | 2 | 0.013 |
| Ageusia | 1 | 0.007 |
| Anaphoresis | 1 | 0.007 |
| Arrhythmia | 1 | 0.007 |
| Axillary pain | 1 | 0.007 |
| Blister | 1 | 0.007 |
| Body temperature increased | 1 | 0.007 |
| Claustrophobia | 1 | 0.007 |
| Coccydynia | 1 | 0.007 |
| Confusional state | 1 | 0.007 |
| Dehydration | 1 | 0.007 |
| Drug hypersensitivity | 1 | 0.007 |
| Dysphonia | 1 | 0.007 |
| Ear congestion | 1 | 0.007 |
| Ear pain | 1 | 0.007 |
| Energy increased | 1 | 0.007 |
| Epigastric discomfort | 1 | 0.007 |
| Eructation | 1 | 0.007 |
| Eye irritation | 1 | 0.007 |
| Flank pain | 1 | 0.007 |
| Irritability | 1 | 0.007 |
| Mental status changes | 1 | 0.007 |
| Movement disorder | 1 | 0.007 |
| Myocardial ischemia | 1 | 0.007 |
| Ocular hyperemia | 1 | 0.007 |
| Oropharyngeal discomfort | 1 | 0.007 |
| Paresthesia oral | 1 | 0.007 |
| Paranasal sinus hypersecretion | 1 | 0.007 |
| Parosmia | 1 | 0.007 |
| Pharyngeal edema | 1 | 0.007 |
| Photopsia | 1 | 0.007 |
| Piloerection | 1 | 0.007 |
| Precordial exam finding | 1 | 0.007 |
| Presyncope | 1 | 0.007 |
| Pupils unequal | 1 | 0.007 |
| Pyrexia | 1 | 0.007 |
| Rosacea | 1 | 0.007 |
| Salivary hypersecretion | 1 | 0.007 |
| Sensation of pressure | 1 | 0.007 |
| Sensory disturbance | 1 | 0.007 |
| Sinus disorder | 1 | 0.007 |
| Speech disorder | 1 | 0.007 |
| Strabismus | 1 | 0.007 |
| Stress | 1 | 0.007 |
| Tension | 1 | 0.007 |
| Tension headache | 1 | 0.007 |
| Thirst | 1 | 0.007 |
| Unresponsive to stimuli | 1 | 0.007 |
| Urinary incontinence | 1 | 0.007 |
| Yawning | 1 | 0.007 |

**Total number of Gilenya Assessment Network clinics patients with available AE data, *n* = 15,015; 10 patients attended the clinic and began the first-dose observation procedure but did not receive a dose of fingolimod. *AE* adverse event. *AV* atrioventricular; *ECG* electrocardiogram.**
